# Avasimibe Abolishes the Efficacy of Fluvastatin for the Prevention of Cancer in a Spontaneous Mouse Model of Breast Cancer

**DOI:** 10.3390/ijms26062502

**Published:** 2025-03-11

**Authors:** Anjana Bhardwaj, Alexander Koh, Rhea Bhala, Janvi Sandhu, Zhenlin Ju, Leslie Faye Cando, Jing Wang, Isabelle Bedrosian

**Affiliations:** 1 Breast Surgical Oncology, The University of Texas MD Anderson Cancer Center, Houston, TX 77030, USA; 2 Department of Bioinformatics, The University of Texas MD Anderson Cancer Center, Houston, TX 77030, USA; 3 College of Medicine, University of the Philippines Manila, Manila 1000, Philippines

**Keywords:** statin, avasimibe, TNBC, drug–drug interaction, mouse, breast cancer, prevention

## Abstract

The cholesterol biosynthesis pathway is upregulated during breast cancer development and progression. Inhibition of the aberrantly upregulated cholesterol pathway by statins reduces breast tumor incidence and burden by 50% in SV40 C3(1) TAg mice, a mouse model of triple negative breast cancer. We hypothesized that fluvastatin’s preventive efficacy could be further enhanced by co-targeting the statin-induced restorative feedback pathways that tightly control the cholesterol pathway and are involved in resistance to statins. Acyl-coenzyme A: cholesterol acyltransferase (*ACAT*)2 is a cholesterol esterification gene that is upregulated in statin-resistant MCF10.DCIS cells, and in mammary tumors of statin-non-responsive SV40 C3(1) TAg mice. In support of this hypothesis, a combination of fluvastatin and avasimibe effectively inhibited the cell growth of statin-resistant MCF10.DCIS cells. However, this combination failed to prevent breast tumor formation in SV40 C3(1) TAg mice. Although avasimibe inhibited fluvastatin-induced *ACAT2* mRNA expression in the breast tissue of the combination-treated mice, confirming that avasimibe effectively hit its target, the fluvastatin and avasimibe combination was completely ineffective in preventing breast cancer in vivo, with approximately 90% of mice developing tumors by 22 weeks, similar to the vehicle control group animals. These findings, along with avasimibe’ s known interactions with CYP450 gene family members, suggest that AVA abrogates the efficacy of fluvastatin through enhanced metabolism of fluvastatin in vivo. The findings reported in this brief communication provide a cautionary note for studies proposing the use of avasimibe in combination therapy for cancer prevention and treatment.

## 1. Introduction

The cholesterol biosynthesis/mevalonate (MVA) pathway plays a central role in normal cellular development and in carcinogenesis. In cancer cells, cholesterol is required as a key building block for the assembly of the cell membranes of rapidly dividing cancer cells, and cholesterol pathway activation is associated with poor prognosis across all subtypes of breast cancer patients [1,2,3]. Relevant to breast tumorigenesis, the breast epithelium in women with atypical hyperplasia, a high-risk state, is known to have high cholesterol levels and an increase in oxidative products of cholesterol (5 beta, 6 beta-epoxide) [4]. Additional laboratory evidence for the role of the cholesterol pathway in breast carcinogenesis comes from numerous studies, including our own, that have suggested that cholesterol pathway genes such as 3-hydroxy-3-methyl-glutaryl-coenzyme A reductase (*HMGCR*) are deregulated in transformed cells and drive the initiation and progression of breast cancer [5]. These observations support strategies to target this pathway for the prevention of breast cancer.

Consistent with the driver role of the MVA pathway in breast cancer, a window-of-opportunity trial showed a cholesterol-lowering drug statin to reduce tumor cell proliferation in a subset of patients [6]. Similarly, our preclinical studies found fluvastatin to prevent breast cancer in 50% of mice in an SV40 C3(1) TAg mouse model of TNBC [7]. We studied transcriptional reprogramming in response to statin treatment in statin-sensitive and statin-resistant mice and cell lines to understand this partial efficacy and the mechanisms of statin resistance. While statin-responsive mice had normal restorative feedback in response to statins, we found statin-resistant breast tumors to have more robust feedback upregulation of the cholesterol pathway genes [8]. This is in line with prior studies that have reported the upregulation of multiple cholesterol biosynthesis pathway genes at baseline and a robust sterol regulatory element-binding protein (SREBP)-mediated restorative feedback response with statin treatment as mechanisms that bypass the effect of statin blockade on cell growth. SREBP is a central modulator of the cholesterol biosynthesis pathway that regulates the transcription of genes encoding for multiple key enzymes of this pathway, including *HMGCR*. Therefore, inhibiting SREBP-mediated feedback mechanisms along with statin inhibition of the pathway could potentiate the cytotoxic effects of statins [2].

Among the genes upregulated in cells resistant to statin therapy, we observed acyl-coenzyme A: cholesterol acyltransferase1 (*ACAT1*, also called *SOAT1*) and acyl-coenzyme A: cholesterol acyltransferase 2 (*ACAT2*) [8]. ACATs convert free cholesterol to cholesteryl esters that are required for storing cholesterol in lipid droplets. The accumulation of cholesteryl esters is correlated with more aggressive breast tumors such as those with a higher histological stage and more Ki67 and ER negativity [9]. Thus, cholesterol esterification in conjunction with cholesterol synthesis and export constitute key mechanisms that are involved in maintaining cholesterol homeostasis. Therefore, we hypothesized that dual targeting with ACAT inhibitor and avasimibe, along with a statin, will provide greater efficacy for the prevention of breast cancer compared to a statin alone. Avasimibe was developed as an anti-atherosclerosis drug in the late 1990s because of its ability to lower cholesterol and inhibit atherosclerosis. Recently, avasimibe has been studied for its anticancer potential. These studies have shown that it exerts anticancer activity through the inhibition of tumor cell proliferation and metastasis via the E2F signaling or Wnt/β catenin pathway in pancreatic cancer cells and glioma cells lines [10,11,12]. Avasimibe has also been reported to increase the efficacy of a Kras-based peptide vaccine by decreasing the presence of inhibitory Tregs, and increase the infiltration of CD8^+^T cells in a KrasLA1 GEMM of lung cancer [13]. It remains to be tested whether avasimibe can increase the efficacy of statins to prevent breast cancer. We hypothesized that dual targeting with a statin and avasimibe would more effectively inhibit the cholesterol pathway and its associated homeostatic mechanisms, thus increasing treatment efficacy.

## 2. Results

### 2.1. Fluvastatin Inhibition of MVA Pathway Causes Upregulation of ACAT2 in Ductal Carcinoma in In Situ MCF10.DCIS Cells

**Figure 1:**
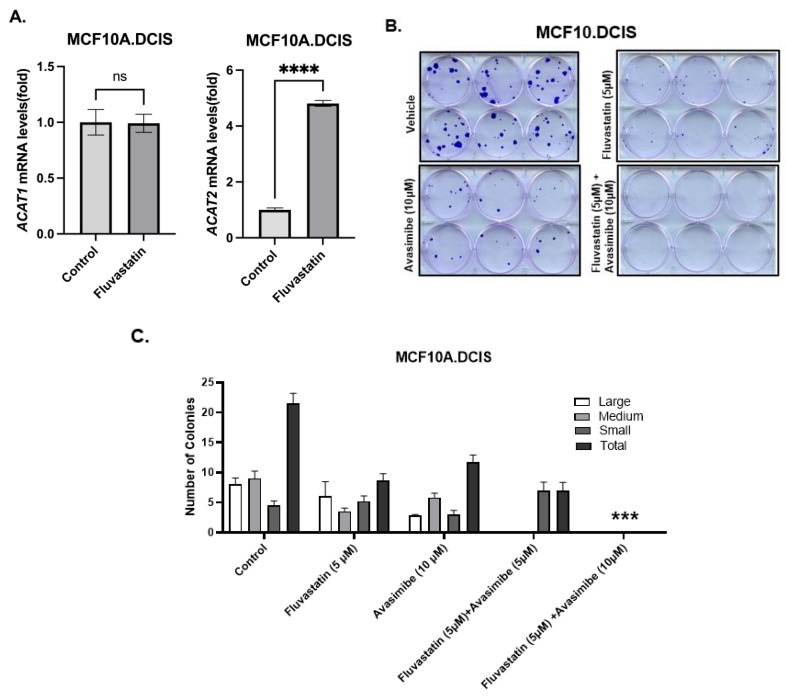
Restorative upregulation of *ACAT2* transcript in fluvastatin-treated MCF10.DCIS and its inhibition with avasimibe sensitizes these cells to fluvastatin. (**A**) Expression of *ACAT1* and *ACAT2* mRNAs in fluvastatin-treated MCF10.DCIS cells according to qPCR. Y axis depicts fold changes in average gene expression that was calculated by using ΔΔCt method after normalizing with ribosomal protein L19. **** indicates significance at *p* < 0.0001, ns indicates no statistical significance. (**B**) Clonogenic survival assay showing crystal-violet-stained colonies formed by MCF10.DCIS cells after 12 days of treatment with fluvastatin, avasimibe, combo, or vehicle control. (**C**) Quantification of colonies formed by MCF10.DCIS cells. Values represent number of colonies (%)  ±  SEM. *** *p*  <  0.001.

Cholesterol pathway targeting by statins is known to trigger restorative transcriptional reprogramming, which plays a role in resistance to statins [2,14]. Resistance to statins, as indicated by the relatively higher expression of cholesterol biosynthesis pathway genes at baseline or as a result of feedback reprogramming, is a feature of inherently statin-resistant cells compared to statin-sensitive cells or can be acquired as a result of long-term exposure to statins. As previously reported, there is a substantial overlap in the gene signature panel that is involved in inherent or acquired resistance to statins [8]. In order to identify gene targets that can be co-targeted to enhance the growth-inhibitory effects of statins, we treated inherently fluvastatin-resistant MCF10.DCIS cells with fluvastatin and measured the levels of *ACAT1/SOAT1* and *ACAT2*, two genes that we previously found to be significantly upregulated in statin-non-responsive mouse breast tumors relative to those in statin-responsive mice (statin-sensitive mammary tissues) [8]. Twenty-four-hour treatment of MCF10.DCIS cells with 10 μM fluvastatin resulted in a 4.8-fold increased expression of *ACAT2* transcripts as assayed by qPCR (Figure 1A, *p* < 0.0001). The upregulation of *ACAT2* transcripts in inherently statin-resistant MCF10.DCIS cells suggests that avasimibe (an ACAT2 inhibitor) could be a potential target for combination with fluvastatin to sensitize these cells.

### 2.2. ACAT2 Inhibition by Avasimibe Sensitizes MCF10.DCIS Cells to Fluvastatin

As a first step towards assessing the growth-inhibitory effects of fluvastatin and avasimibe dual targeting, a colony formation assay (CFA) was performed in inherently statin-resistant MCF10.DCIS cells [8]. These cells were treated with fluvastatin alone (5 μM), avasimibe alone (10 μM and 15 μM), or a combination of fluvastatin and avasimibe for 12 days. Treatment with avasimibe alone (10 μM) caused about a 45% reduction in the total number of cell clones, whereas fluvastatin treatment (5 μM) at half of the dose of avasimibe (10 μM) caused a 60% reduction in the total number of cell clones (Figure 1B). The growth-inhibitory effects of fluvastatin were further potentiated by the addition of avasimibe, as this combination (5 μM fluvastatin and 10/15 μM avasimibe) was able to completely abolish the colonizing ability of MCF10.DCIS cells. No surviving cell clones were observed after 2 weeks of treatment (100% inhibition, *p* < 0.001), (Figure 1B,C). This in vitro sensitization of statin-resistant cells supports our hypothesis that fluvastatin and avasimibe is a rational drug combination.

### 2.3. Avasimibe Treatment Abolishes Chemopreventive Efficacy of Statins to Inhibit TNBC in SV40 C3(1) TAg Mouse Model

**Figure 2:**
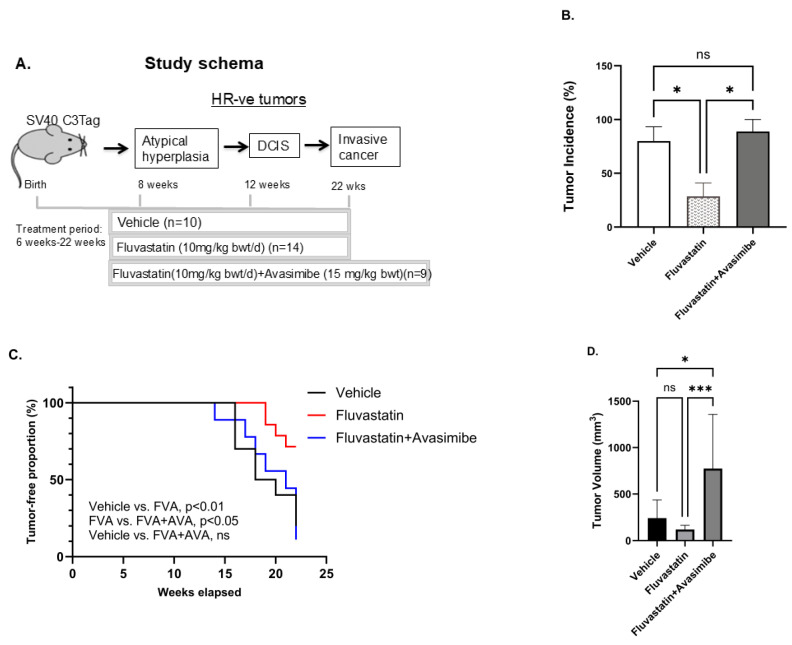
Avasimibe treatment fails to sensitize SV40 C3 TAg mice that develop TNBC to fluvastatin. (**A**) Schematic showing schedule of drug treatment in SV40 C3 TAg mouse model of breast cancer progression. Fluvastatin treatment reduces tumor incidence, delays onset of tumors, and inhibits tumor growth in SV40 C3 TAg mouse model of breast cancer progression, and avasimibe treatment abolishes these protective effects. (**B**) Percentage of mice that developed mammary tumors as determined by macroscopic lesions at time of necropsy at 22 weeks of age. (**C**) Average age at which tumors appear, as shown here as % incidence of palpable-tumor-bearing mice during study, relative to the vehicle control group. (**D**) Average tumor volume in mice that developed mammary tumors as measured using Vernier Calipers at 21–22 weeks of age; * *p*  <  0.05, *** *p* < 0.001, ns = no statistical significance.

To further assess the ability of avasimibe to improve the chemopreventive efficacy of fluvastatin in a mouse model of TNBC, we performed drug efficacy studies in an SV40 C3(1) TAg spontaneous mouse model of TNBC with fluvastatin alone and in combination with avasimibe. Six-week-old SV40 C3(1) TAg mice were treated with either a vehicle, or with fluvastatin alone (10 mg/kg b.wt./d) or in combination with an every-other-day dose of avasimibe (20 mg/kg b.wt.) for 16 weeks until the age of 22 weeks (Figure 2A). To our surprise, and contrary to our hypothesis, we found this combination treatment to render fluvastatin completely ineffective in preventing breast cancer in vivo. Eighty percent of mice developed tumors by 21 weeks of age in vehicle-treated mice, whereas tumor incidence was remarkably low in fluvastatin-treated mice, as only 29% of mice developed tumors in the group treated with fluvastatin alone (Figure 2B, *p* < 0.05). However, the addition of avasimibe to the fluvastatin treatment reduced the efficacy of fluvastatin, with mammary tumor incidence in the combination group rising to 89% of mice (Figure 2B, *p* < 0.05), similar to the vehicle control group. Similarly, the fluvastatin- and avasimibe-treated mice showed earlier onset of tumors at 13 weeks, earlier than the onset in vehicle-treated animals (15 weeks), whereas fluvastatin treatment delayed tumor onset to 19 weeks of age (Figure 2C). The median tumor-free survival of the vehicle-treated animals was 19 weeks, whereas fluvastatin significantly prolonged tumor-free survival, with median tumor-free survival not reached at 22 weeks, when all the animals were sacrificed (*p* < 0.01). On the contrary, the addition of avasimibe to fluvastatin negated the disease-free survival benefits observed with fluvastatin alone, as mice receiving the combination treatment had a median disease-free survival of 21 weeks, comparable to the vehicle treated mice (21 weeks vs. 19 weeks, *p* value = 0.991). In addition to reducing tumor incidence and extending tumor-free survival, fluvastatin treatment led to a 30% decrease in the average tumor volume of the mice that developed tumors (from 100 mm^3^ to 30 mm^3^, ns = no statistical significance, Figure 2D). In contrast, mice receiving the combination treatment had a mean tumor volume of 120 mm^3^, comparable to that of vehicle-treated animals (Figure 2D). These indicators of anti-tumor activity indicate that the combination of avasimibe and fluvastatin is not an effective cancer-preventive strategy in vivo.

### 2.4. Effect of Drug Treatments on Biomarker (ACAT1 and ACAT2) Expression

**Figure 3:**
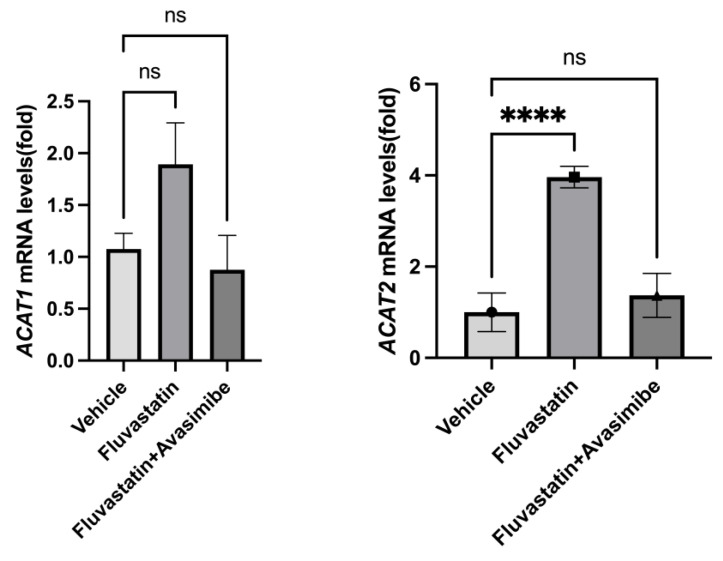
Avasimibe treatment inhibits fluvastatin-induced *ACAT2* in mammary tissue of SV40 C3 TAg mice. Expression of *ACAT1* and *ACAT2* mRNAs in mammary glands of SV40 C3 TAg mice according to qPCR. Y axis depicts fold changes in average gene expression that was calculated by using ΔΔCt method after normalizing with ribosomal protein L19. **** indicates significance at *p* < 0.0001, ns = no statistical significance.

In order to understand the failure of avasimibe treatment to prevent breast cancer in SV40 C3(1) TAg mice, we tested whether avasimibe hit its target and inhibited ACAT1 and ACAT2 expression. As expected, fluvastatin-treated mouse mammary glands showed an induction in both *ACAT1/SOAT1* and *ACAT2* (2.4-fold and 3.96-fold) relative to the vehicle control group (Figure 3, *p* < 0.01, *p* < 0.001). This is in line with the restorative feedback upregulation of *ACAT*s by statins [8]. However, when treated with the combination of fluvastatin and avasimibe, the expression levels of *ACAT2* were not significantly different from those seen in animals treated with a vehicle control, confirming that in the combination-treated animals, avasimibe effectively inhibited the expression of ACAT2 transcripts in breast tumors (Figure 3). The tested dose of avasimibe (10 mg/kg b.wt.) that was administered every other day in combination with fluvastatin did not cause a significant reduction in *ACAT1* mRNA levels, which is in line with avasimibe’s approximately 3-fold lower IC50 for *ACTA2* inhibition relative to *ACAT1* inhibition. Together, this suggests that the combination treatment of fluvastatin and avasimibe, at the chosen dose and route, was successful at inhibiting the expression of breast *ACAT2*.

Since avasimibe is a known inducer of CYP450 activity [15,16], an enzyme that is involved in metabolizing more than 70% of drugs in the body, we postulated that the abrogation of fluvastatin efficacy when combined with avasimibe is likely due to avasimibe’s induction of CYP450 with the consequent enhanced metabolism of fluvastatin [15,16,17]. We assayed *CYP* gene levels in the mammary glands of mice treated with fluvastatin alone and in combination with avasimibe using qPCR. These analyses showed that the combination had no effect on the expression of several key *CYP* gene superfamily members (*CYP2B10*, *CYP2B19*, *CYP2C37*, and *CYP3A11*) and other genes involved in efflux pumps (*NR1I2/PXR* and *ABCC1*; Figure S1). These negative findings may be attributed to the presence of large number of *CYP* pseudogenes in the murine *CYP* gene superfamily, which can interfere with gene quantification in PCR-based assays [18].

Taken together, our data suggest that despite the appropriate inhibition of *ACAT2,* the known effects of avasimibe on CYP450 activity enhanced fluvastatin metabolism and negated any benefit to dual targeting of the cholesterol synthesis pathway for breast cancer prevention.

## 3. Discussion

The cholesterol pathway is often deregulated during breast tumorigenesis, and as such, targeting this pathway offers an attractive strategy for prevention. However, strong activation of statin feedback mechanisms will drive the upregulation of genes, such as *ACAT*s, that can drive resistance to statin treatment. We therefore assessed the efficacy of combining an ACAT2 inhibitor, avasimibe, with fluvastatin in a mouse model of TNBC. Despite the combination showing promising anticancer effects in vitro, avasimibe completely abolished the chemopreventive efficacy of fluvastatin in vivo, likely due to the effects of avasimibe on drug clearance [15,16,18]. Studying drug metabolism and drug interactions requires complex cell and tissue interactions that are not present in cell lines. Additionally, immortalized cell lines express low levels of drug metabolism and drug efflux genes [19,20]. Hence, it was not completely surprising that, although avasimibe sensitized DCIS cells to fluvastatin in vitro, it rendered fluvastatin completely ineffective in vivo. These results further add to the concerns that led to the halting of avasimibe in phase III clinical trials in atherosclerosis. While avasimibe showed a good safety profile and passed phases I and II of the clinical trial, it was found to lower the absorption of co-administered drugs [21].

Since the clinical trial of avasimibe in atherosclerosis in the late 1990s, there has been a renewed interest in avasimibe as a potential anticancer agent as a result of data showing that avasimibe can potentiate the anti-tumor response of CD8+T cells [22]. This pioneering study by Yang et al. in a *Nature* publication led to multiple subsequent studies testing avasimibe alone or in combination with immune vaccines [22]. These in vivo studies, where avasimibe was tested as a single agent, found avasimibe to inhibit the growth of various cancer cells, including glioma, pancreatic cancer, hepatocellular carcinoma, Lewis lung carcinoma, and breast cancer [10,11,12]. Since avasimibe was found to potentiate the function of CD8+T cells through the suppression of CD8+ T cell exhaustion, a fairly large number of studies have tested avasimibe and found it to be synergistic with multiple immune therapies such as peptide vaccines, DC vaccines, and CAR-T cell therapy [13,23,24]. These studies have propelled an interest in taking avasimibe to clinical trials to identify its potential for use as a combination agent with immune therapies for both the prevention and treatment of cancer.

However, avasimibe is known to cause significant drug interactions through the direct activation of human pregnane X receptor (*hPXR*), the subsequent induction of its target genes, such as *CYP450 3A4 (CYP3A4)* and multiple drug resistance protein 1, and the inhibition of *CYP2C9* [15,16]. As such, these effects of avasimibe on drug clearance may outweigh its potentially positive effects on immune pathways, as underscored by our data showing that avasimibe negated the preventative efficacy of an orally co-administered drug—fluvastatin. We believe that avasimibe reduced the bioavailability of fluvastatin, a lipophilic statin that, as such, has a small half-life (1–3 h), and caused fluvastatin treatment failure. Fluvastatin is known to be primarily metabolized by CYP2C9 and to some extent by CYP2C8 in humans. However, the genomic organization of the *CYP2C* gene cluster is significantly more complex in mice, where 15 functional genes are described as a part of this cluster compared with only 4 genes in human [18]. Because of these differences in multiplicity and also in sequence variation, it is not possible to define orthologous genes between these two species. These challenges, combined with the large number of pseudogenes in murine *CYP* genes, prevented us from finding any changes in the expression of the *CYP* gene family by qPCR. Avasimibe is known to regulate *CYP* gene expression through the induction of human pregnane X receptor (*hPXR or NR1I2*) [15]. Once activated, *PXR* increases the expression of its target gene—multiple drug resistance protein 1 (*MRP1/ABCC1*) [15]. However, due to significant species differences in ligand binding selectivity between humans and mice for *PXR* [25], studying the clinically relevant role of PXR-regulated genes (such as *CYP*) in mice requires the creation of humanized mice. While directly assaying CYP450 activity through enzymatic assays could have determined if avasimibe rendered fluvastatin ineffective through the activation of CYP450, these assays required fresh tissues, which were not available in our study.

This brief communication suggests that the ancillary effects of avasimibe will likely limit its efficacy and potential utility to patients. More than 60% of the commonly used drugs, such as antibiotics, antidepressants, benzodiazepines, statins, and calcium channel blockers, are metabolized through CYP3A4 [26]. Therefore, a closer evaluation of trade-offs will have to be conducted when prescribing avasimibe in combination with immune therapies to patients. Whether other strategies can be utilized to improve the chemopreventive efficacy of fluvastatin remains uncertain, but strategies such as CRISPR screening [27] could be utilized to identify drug resistance targets that could be potentially targetable for this purpose.

## 4. Materials and Methods

### 4.1. Cell Lines

MCF10.DCIS, a ductal carcinoma in situ cell line, was obtained from Wayne State University, Detroit, MI, USA. The cells were grown in antibiotic free, glutamine-containing DMEM/Ham’s F12 (50:50) media supplemented with 5% horse serum, CaCl_2_ (1.05 mM), and HEPES (10 mM). The cells were used within the first 10 passages after being obtained from the vendor. All reagents were bought from Thermo Fisher Scientific, Waltham, MA, USA, unless specified otherwise. 

### 4.2. Mice and Drug Treatments

SV40 C3(1) TAg transgenic female mice that spontaneously develop triple negative breast tumors were obtained from JAX labs, Bar Harbor, ME, USA. These mice go through various stages of breast cancer progression prior to forming invasive tumors at the age of 22 weeks. For this chemoprevention study, mice were randomly subdivided into different categories of drug treatments (fluvastatin alone, avasimibe+ fluvastatin, and vehicle control) that started at the age of 6 weeks, prior to the activation of the oncogenic SV40T antigen transgene, and lasted until 22 weeks of age. Fluvastatin was continuously administered to the mice via drinking water at a dose of 10 mg/kg b.wt./d. Fluvastatin-containing water was changed every other day. The water consumption per mouse cage was recorded twice a week, and fluvastatin concentration in the water was adjusted to maintain the desired dose of fluvastatin. Avasimibe was administered to mice by oral gavage every other day, 3 times a week, at a dose of 15 mg/kg b.wt./d. Avasimibe solution was prepared by dissolving the drug in DMSO (0.5% DMSO in PBS) and then further diluting it with a solution of Tween-80 (0.1%); carboxy-methylcellulose sodium salt (0.1%), followed by sonicating for 10 min at 4 °C. The doses of both fluvastatin and avasimibe were well within the dose range that is relevant to humans. 

### 4.3. Tumor Onset, Tumor Incidence, and Tumor Volume

The onset of mammary tumors was tracked by gross palpation of the cervical, abdominal, and inguinal mammary glands in mice starting at the age of 10 weeks and continued until 22 weeks of age. A mouse with one or more palpable tumors larger than 3 mm was considered tumor-bearing for the tumor onset studies. Tumor incidence was calculated based on the number of macroscopic tumors discovered in one or more mammary glands of mice at the time of necropsy. Tumor volume (in mm^3^) was measured by gross palpation of mouse mammary glands using Vernier calipers starting at the age of 15 weeks and continued until 21 weeks of age. Tumor length and width were measured and used to calculate tumor volume using the following formula: Tumor volume = (Length × Width^2^)/2.

### 4.4. qPCR

The expression of *ACAT1/SOAT1*, *ACAT2*, *CYP2B10*, *CYP2B19*, *CYP2C37*, *CYP3A11*, *NR1I2 (PXR)*, and *ABCC1* transcripts was measured by first generating cDNA using the Iscript reverse transcriptase (RT) kit (BioRad, Hercules, CA, USA). qPCR was performed as described in [5]. Briefly, the RT reaction was mixed with iQ SYBR Green supermix (BioRad, Hercules, CA, USA) and primer pairs using a Thermocycler. We ordered predesigned inventoried KiCqStart™ Primers SYBR^®^ Green Primers from Sigma-Aldrich (St. Louis, MO, USA) to detect the transcripts of interest by qPCR. The PCR amplicons were quantified using the Ct value of the gene of interest normalized against the Ct of the ribosomal protein *RPL19* gene.

### 4.5. Colony Formation Assay

A colony formation assay was performed by plating 100 cells per well in a 6-well plate and treating them with the indicated concentrations of fluvastatin, avasimibe, and the combination for 12 days, after which the colonies were stained with crystal violet as described previously [5]. Colony formation is a cumulative measure of the ability of cells to colonize without cell-to-cell contact and cell proliferation. While the size of clones suggests the ability of cells to proliferate, the total number of colonies suggests the relative number of cells that survive without cell-to-cell contact. In order to capture the effects of drugs on both cellular processes, we present a separate count of large (>2.5 mm), medium (1.5–2.5 mm), and small (1–1.5 mm) colonies. Although the total number of colonies might seem relatively similar in some treatment groups, a significantly lower number of large and medium colonies suggested a stronger effect of the drugs on cellular proliferation.

### 4.6. Statistics

A minimum of nine mice per group provided more than 80% power to detect the 2-fold difference between the two groups. Student’s unpaired t test or one-way ANOVA was applied to determine the statistical difference between the two groups. The statistical significance between tumor-free survival in each group was calculated using a Chi Square test. *p* values < 0.05 were considered significant.

## 5. Conclusions

Drug combinations that seem rational from in vitro studies need to be tested in preclinical model systems prior to testing in clinical trials. Our study reports that avasimibe completely abolished the cancer-preventive efficacy of fluvastatin in a mouse model of triple negative breast cancer, despite the combination showing promising anticancer efficacy in vitro. We believe this was likely due to ancillary effects of avasimibe that resulted in faster clearance of fluvastatin. Our findings raise concerns regarding studies proposing the use of avasimibe for cancer prevention and treatment.

## Data Availability

All of the data supporting the results reported in this article are presented in the form of figures in this manuscript.

## Supplementary Materials

The supporting information can be downloaded at: https://www.mdpi.com/article/10.3390/ijms26062502/s1.

## Abbreviations

SV40 C3(1) TAg: Simian Virus 40 C3(1) large T-antigen; DCIS: ductal carcinoma in situ; TNBC: triple negative breast cancer; ACAT: acyl-coenzyme A: cholesterol acyltransferase; MVA: mevalonate; HMGCR: 3-hydroxy-3-methyl-glutaryl-coenzyme A reductase; SREBP: sterol regulatory element-binding proteins; SOAT1: Sterol-O-Acyl transferase 1.
