# Supplementary material for: Avasimibe Abolishes the Efficacy of Fluvastatin for the Prevention of Cancer in a Spontaneous Mouse Model of Breast Cancer
Source: Int J Mol Sci. 2025 Mar 11;26(6):2502. doi: 10.3390/ijms26062502 (PMC11942263; doi:10.3390/ijms26062502)
Supplement: Supplementary file 1 [file ijms-26-02502-s001.zip › ijms-3467286-supplementary.pptx]

## Slide 1
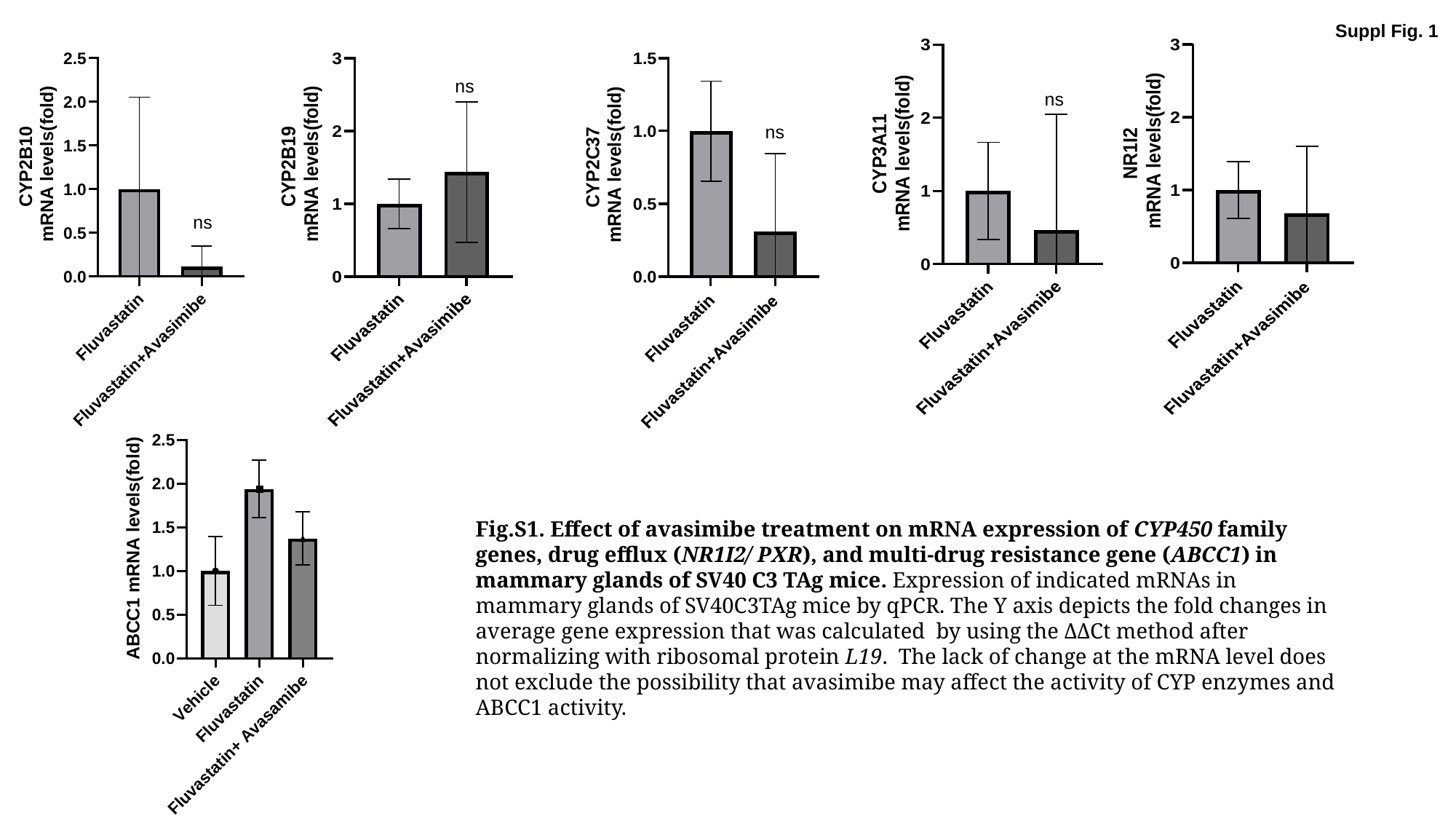

Suppl Fig. 1
ns
ns
ns
ns
Fig.S1. Effect of avasimibe treatment on mRNA expression of CYP450 family genes, drug efflux (NR1I2/ PXR), and multi-drug resistance gene (ABCC1) in mammary glands of SV40 C3 TAg mice. Expression of indicated mRNAs in mammary glands of SV40C3TAg mice by qPCR. The Y axis depicts the fold changes in average gene expression that was calculated by using the ΔΔCt method after normalizing with ribosomal protein L19. The lack of change at the mRNA level does not exclude the possibility that avasimibe may affect the activity of CYP enzymes and ABCC1 activity.
